# A Source of Riches

**Published:** 1899-10

**Authors:** 


A SOURCE OF RICHES, consisting of large tracts of forest with abundance of rubber trees, is said to exist in the unexplored interior of the State of Bahia (Brazil), but unfortunately, owing to the difficulties of transport through a country without roads, and to the fact that the forests are inhabited by different tribes of Indians, some of whom are cannibals, it is said to be unavailable. The quantity of rubber produced in the State during 1898, however, increased materially, the high prices realized inducing collectors to proceed farther afield for supplies.—*Pharmaceutical Journal.*

